# Neurosurgical Management of Pott’s Puffy Tumor in an Obese Adolescent with Asthma: Case Report with a Brief Review of the Literature

**DOI:** 10.7759/cureus.2836

**Published:** 2018-06-19

**Authors:** Guilherme G Podolsky-Gondim, Marcelo V Santos, Vinícius M Carneiro, Lucas Pires Augusto, Romilto Da Costa Pacheco Neto, Ricardo Santos de Oliveira

**Affiliations:** 1 Division of Neurosurgery, Department of Surgery and Anatomy, Ribeirão Preto Clinics Hospital, Ribeirão Preto Medical School, University of São Paulo, Ribeirão Preto, BRA; 2 Division of Pediatric Neurosurgery, Department of Surgery and Anatomy, Ribeirão Preto Clinics Hospital, Ribeirão Preto Medical School, University of São Paulo, Ribeirão Preto, BRA; 3 Division of Pediatric Neurosurgery, Department of Surgery and Anatomy, Ribeirão Preto Clinics Hospital, Ribeirão Preto Medical School, University of São Paulo, Ribeirao Preto , BRA

**Keywords:** frontal sinusitis, pott puffy tumor, epidural abscess

## Abstract

Pott’s puffy tumor is a rare and severe complication of frontal sinusitis, characterized by the progressive swelling of the frontal soft tissues secondary to a subperiosteal abscess. Radiological imaging with ultrasound, computed tomography (CT), and magnetic resonance imaging (MRI) are important diagnostic tools in establishing diagnosis and treatment planning. Early surgery along with intravenous antibiotics are required in order to achieve a good recovery. The authors report a case of Pott’s puffy tumor in an obese 14-year-old male, with a previous history of asthma and a chronic use of steroids, treated with neurosurgical debridement followed by a combined course of intravenous (IV) and oral antibiotics, who had a favorable outcome upon long-term follow-up. In addition, a brief review of the current medical literature was performed for a discussion on the diagnostic and therapeutic features of this pathology.

## Introduction

More than 250 years have passed since the British surgeon Sir Percival Pott initially described what is now known as the Pott’s puffy tumor, a subperiosteal abscess of the frontal bone presenting as a progressive forehead edema secondary to frontal osteomyelitis following frontal sinusitis [1-2]. Although a rare clinical complication in the modern area of antibiotics, there are still recent reports of cases in adult and pediatric patients [1-13].

We describe herein a case of a male adolescent diagnosed with a Pott’s puffy tumor with an epidural abscess in addition to the classical frontal subperiosteal abscess and discuss the literature regarding the diagnosis and treatment of this pathology.

## Case presentation

A 14-year-old adolescent was referred to the emergency department (ED) for a neurosurgical consultation with a chief complaint of progressive left forehead bulging in the last three weeks. His parents reported that the patient was seen by a general physician in another institution at the beginning of the symptoms and received a course of oral antibiotics (amoxicillin and clavulanic acid) for the past 21 days. Nevertheless, due to a worsening of the edema on his forehead, CT and ultrasound scans of the head were performed and the patient was referred for a neurosurgical assessment. The patient had a history of asthma, with the daily use of nasal formoterol and budesonide and the chronic use of oral prednisone monthly for up to two to three days. He had recently been referred for a pediatric endocrinological evaluation due to marked obesity (body mass index of 45) with Acanthosis nigricans. There were no reports of head trauma, previous surgery, or hospital admissions.

**Figure 1:**
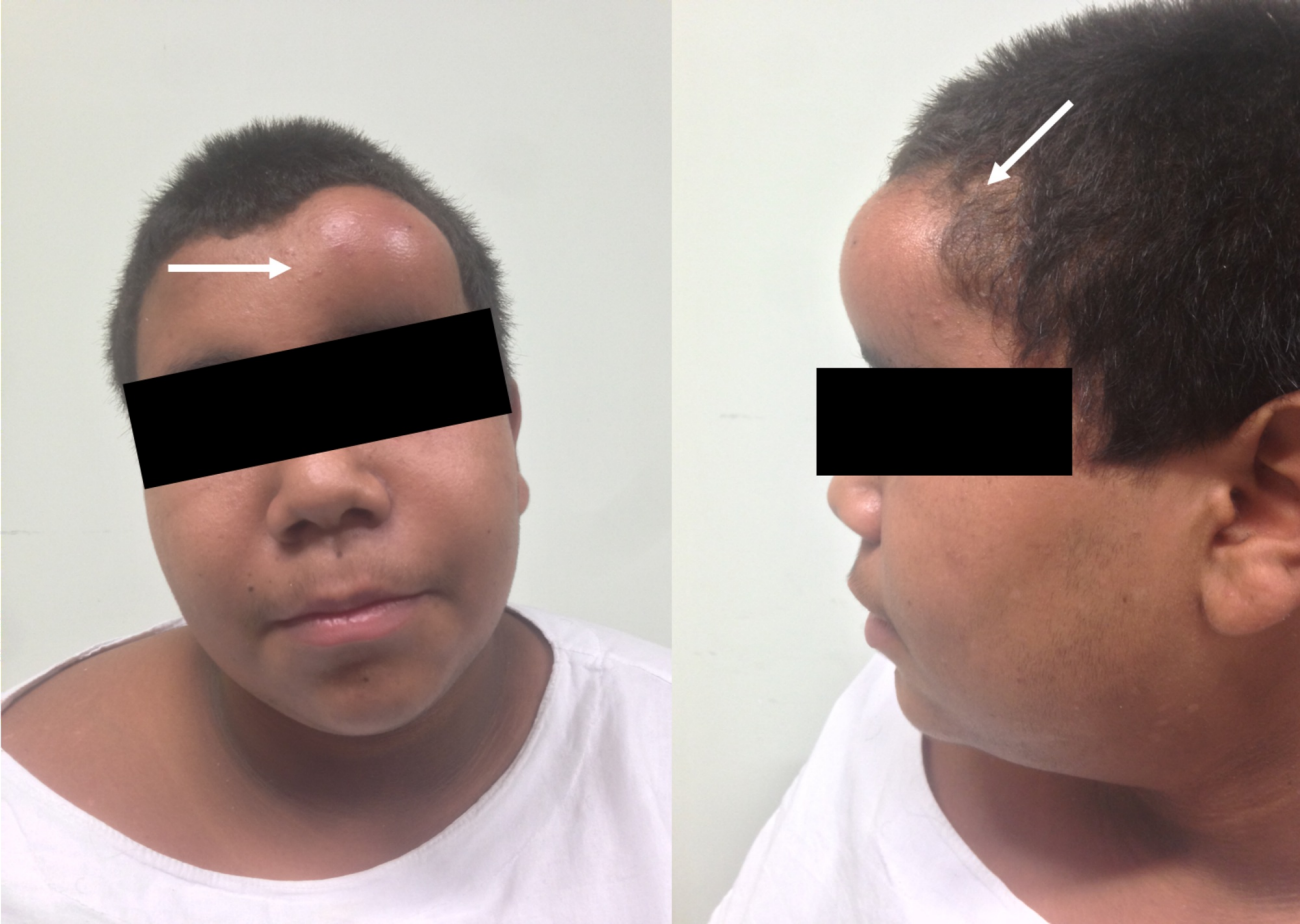
Preoperative photographs Note the soft-tissue swelling in the left frontal area (white arrow).

Upon physical examination, marked obesity was noted with Acanthosis nigricans and a compressible soft-tissue swelling in the left forehead (Figure 1), with an otherwise unremarkable neurological exam.

**Figure 2:**
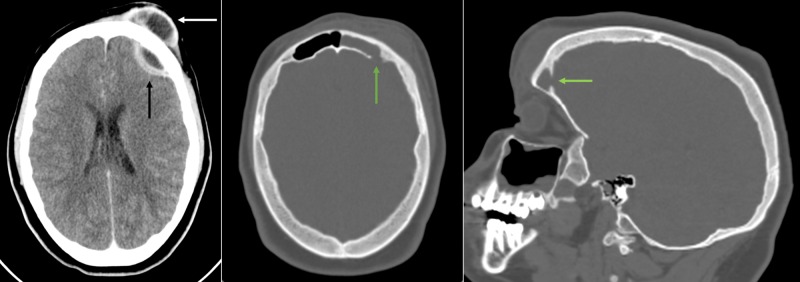
Preoperative CT scans of the head Left: Contrast-enhanced axial computed tomography (CT) scan with epidural (black arrow) and frontal periosteal (white arrow) enhancing mass compatible with an abscess. Middle: Note the bony erosion of the internal wall of the frontal sinus (green arrow) seen on the axial bone window CT scan. Right: Parasagittal imaging of the bony erosion (green arrow) seen in the middle scan.

**Figure 3:**
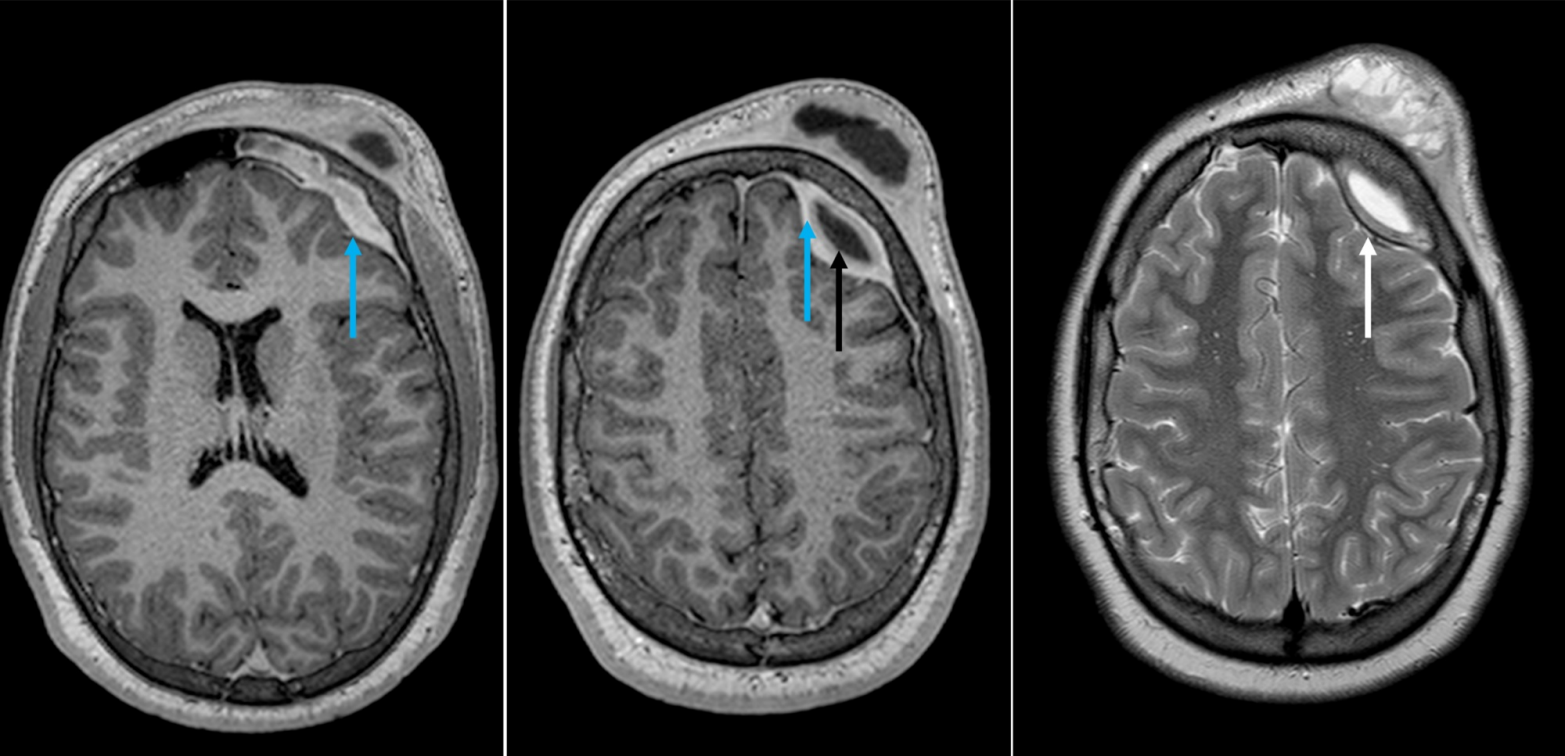
Preoperative MRI of the head: axial reconstruction Preoperative gadolinium-enhanced MRI of the head. Left and middle: An epidural contrast-enhanced capsule (blue arrow) with a center with low signal on a T1-weighted sequence was seen (black arrow), compatible with an abscess. Right: Note the dura mater (white arrow) interposed between the left frontal lobe and the epidural mass with a hyperintense signal on T2-weighted sequence. MRI: magnetic resonance imaging

**Figure 4:**
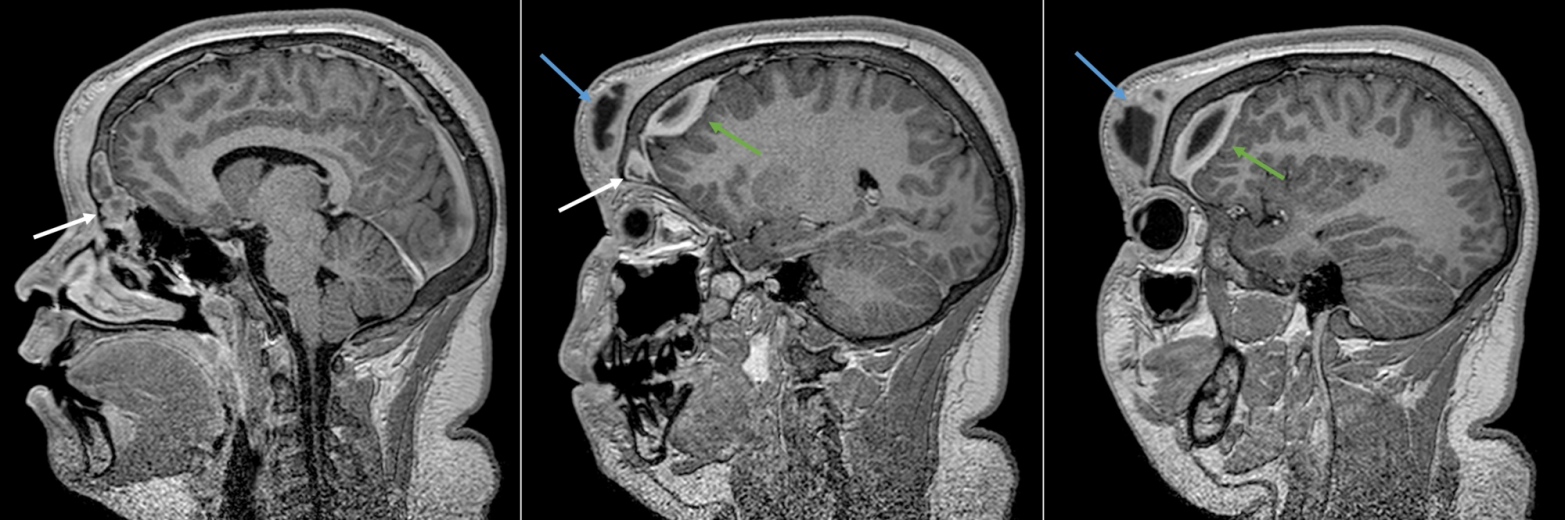
Preoperative MRI of the head: parasagittal reconstruction Parasagittal slices of the preoperative gadolinium-enhanced magnetic resonance imaging (MRI) of the head. In the left and middle images, there is an enhancing lesion filling the left frontal sinus (white arrow) and extending both epidurally (middle and right images - green arrow) and to the periosteum (blue arrow).

Radiological imaging with contrast-enhanced CT and gadolinium-enhanced magnetic resonance scans of the head were performed, showing extensive frontal-ethmoidal sinusitis with bone erosion of the frontal sinus internal and external walls and an epidural and periosteal left-frontal mass with capsular contrast-enhancement suggestive of an abscess (Figures 2-4).

**Figure 5:**
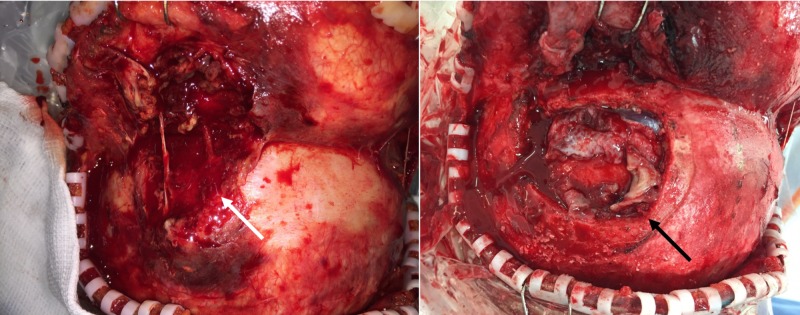
Intraoperative photographs Intraoperative photograph after the bifrontal flap was reflected anteriorly, demonstrating the extensive periosteal and subcutaneous soft tissue compromise (white arrow) and, after left-side frontal craniotomy, showing the epidural mass and debris (black arrow).

After parental consent, the patient was submitted to surgery with a bicoronal skin incision, exposing the infiltrated subcutaneous tissue. After a left frontal craniotomy, an epidural mass compatible with an abscess was found (Figure 5).

**Figure 6:**
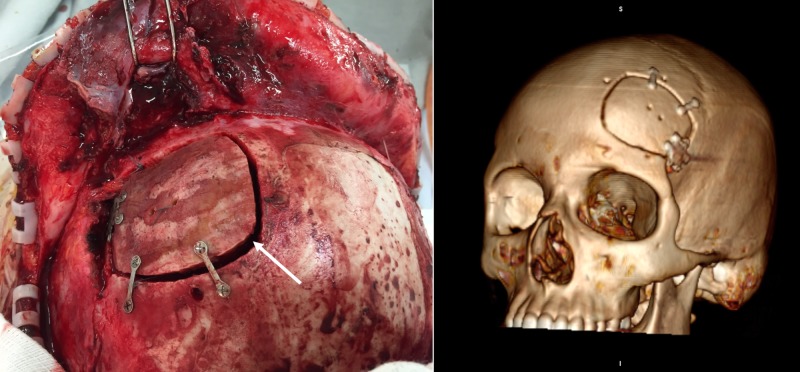
Final surgical aspect of the craniotomy Left: Intraoperative view of the final surgical aspect after fixation of the bone flap (white arrow) with titanium plates and screws. Right: 3D reconstruction of the postoperative computed tomography (CT) scan of the head.

The left frontal sinus was cranialized with an exenteration of the mucosa in order to avoid late complications, such as mucocele formation, and after an extensive removal of debris and specimen sampling, a pedicled pericranium flap was inserted in the frontal sinus drainage pathway and the bone flap was fixed (Figure 6).

**Figure 7:**
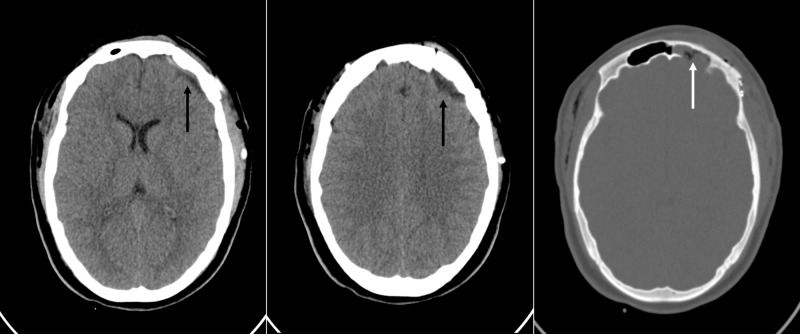
Postoperative CT scans of the head Postoperative contrast-enhanced computed tomography (CT) of the head. Note in the left and middle images the absence of a residual lesion (black arrow) on the axial slices with the brain window and, in the right image, with the bone window, the post-operative cranialization of the left frontal sinus with the filling of the cavity with a periosteum graft (white arrow).

The postoperative period was uneventful, and the patient was submitted to a four-week course of intravenous ceftriaxone/ oxacillin/metronidazole followed by four weeks of oral amoxicillin with clavulanic acid after discharge, as discussed with the Institutional Commission for Control of Infectious Diseases, based on the favorable clinical response of the patient. A post-operative contrast-enhanced CT scan showed no signs of complications or residual lesions (Figure 7).

The culture of the intraoperative lesion was positive for Peptostreptococcus species. The patient was followed in the pediatric neurosurgical outpatient clinic, with no signs of recurrence. However, 18 months after surgery, the patient was referred for an otorhinolaryngologist consultation following chronic sinusitis.

## Discussion

Pott’s puffy tumor is a rare entity; however, there is an increasing number of reports in the medical literature in recent years, particularly in the pediatric population, which may be attributed to delayed or inadequate treatment of frontal sinusitis [2]. Clinical signs include, in the majority of cases, frontal swelling, fever, and headache [2-4]; however, less frequently, there are reports of neurological symptoms, such as focal seizures, cranial nerve or motor deficits [5], a sinocutaneous fistula [6], and eyelid necrosis [7].

Differential diagnosis for any swelling in the forehead includes, but is not limited to, soft-tissue infection, such as carbuncle and infected sebaceous cyst; benign tumors, such as dermoid cysts, lipomas, intraosseous lipomas, lipoblastomas, frontal sinus mucoceles, and superficial temporal artery pseudoaneurysms, and, finally, malignant tumors, such as metastases or aggressive frontal meningiomas [8-9].

The diagnostic workup should include contrast-enhanced CT scan with brain and bony sequences to investigate the presence of an epidural or subdural abscess and to estimate the extent of bone erosion. Also, gadolinium-enhanced MRI may help define the involvement of brain parenchyma or dura mater [3]. Pneumocephalus also may be found [10]. Ultrasound imaging of the forehead may assist in the initial radiological investigation and to guide needle specimen collection for culture [11]. 

Common causative organisms are Streptococcus species (alpha- and beta-hemolytic Streptococci and Peptostreptococci), Hemophilus influenza*, *anaerobic bacteria, such as Fusobacterium species and Bacteroides species, and, less commonly, Staphylococcus aureus and Enterococci species [2].

In our case, the patient was referred after an initial clinical suspicion of soft-tissue frontal infection and was shown by the CT and MRI scans to present an epidural abscess associated with frontal osteomyelitis with the erosion of the inner table of the left frontal sinus, although no neurological symptoms were present upon admission. Also, in our case, we wondered if the chronic use of corticoids due to asthma and the association of obesity had any relation with the occurrence of this rare complication; however, we couldn’t find any previous reports of similar clinical scenarios.

After diagnosis, the management should include broad-spectrum antibiotics and the surgical evacuation of the abscess, which may be performed via open surgery [1-2,7] or, more recently, through minimally invasive techniques (i.e. frontal sinus endoscopic surgery) [12-13]. There are reports of conservative treatment with broad-spectrum antibiotics without surgery; however, a close follow-up with repeat imaging should be considered in these cases [3].

Prognosis, in general, is favorable, even if neurological symptoms are present upon admission; obviously, prompt recognition and treatment are mandatory for successful outcomes.

## Conclusions

Although sometimes depicted as a rare occurrence in the age of broad-spectrum antibiotics, Pott’s puffy tumor must be suspected as a serious complication of frontal sinusitis in both pediatric and adult patients. Early clinical suspicion, diagnosis, and the treatment of the frontal sinusitis and its complications with surgery and broad-spectrum intravenous antibiotics bring up the possibility of good recovery.
